# Magnetite layer formation in the Bushveld Complex of South Africa

**DOI:** 10.1038/s41467-022-28000-9

**Published:** 2022-01-20

**Authors:** Zhuosen Yao, James E. Mungall

**Affiliations:** 1 State Key Laboratory of Geological Processes and Mineral Resources and School of Earth Resources, China University of Geosciences, 430074 Wuhan, China; 2 Department of Earth Sciences, Carleton University, Ottawa, ON K1S 5B6 Canada

**Keywords:** Geochemistry, Petrology

## Abstract

The great economic significance of layered mafic-ultramafic intrusions like the Bushveld Complex of South Africa results from the existence within them of some layers highly concentrated in valuable elements. Here we address the origins of the Main Magnetite Layer, a globally important resource of Fe-Ti-V-rich magnetite. Previous models of in situ fractional magnetite crystallization require frequent ad hoc adjustments to the boundary conditions. An alternative model incorporating compositional convection near the top of the pile and infiltration of the pile from beneath by migrating intercumulus melt fits observations without any adjustments. Lateral variations in Cr concentration formerly held as indisputable evidence for in situ crystallization can be accommodated better by models of reactive melt infiltration from below. The choice of models has pivotal ramifications for understanding of the fundamental processes by which crystals accumulate and layers form in layered intrusions.

## Abstract (web summary)

The Main Magnetite Layer of the Bushveld Complex of South Africa is an economically important deposit of Fe, Ti and V but its mode of formation is enigmatic. Models of fractional crystallization and reactive transport show that it probably accumulated as a loose mush and subsequently was compacted rather than forming in situ.

## Introduction

Because it is impossible to observe directly the processes that produce layered plutonic mafic rocks, the nature of these processes must be inferred from the record of composition, mineral mode, and texture of the final products of emplacement, crystallization, and cooling of the magma^1^. Even the most basic of questions, such as whether or not crystals form within the melt and settle to the floor^2,3^ or are nucleated and grow in situ (i.e., by heterogeneous nucleation in direct physical contact with the solid base)^4,5^, remain open to controversy after a century of dedicated research effort. Progress will be difficult without the resolution of such fundamental questions. A common approach to attempt to resolve these matters is to produce a forward geochemical or physical model and use it to demonstrate the feasibility of a particular hypothetical process; however, there are many examples of cases where totally contradictory hypotheses can be framed in forward models that successfully match observations^6,7^. The resolution of these questions is of more than purely academic interest because some of the layers in question contain mineral deposits of global economic significance^8^. It is therefore essential to address the larger framework within which individual hypotheses are framed before we can make reasoned judgments about which ones to favor.

**Fig. 1:**
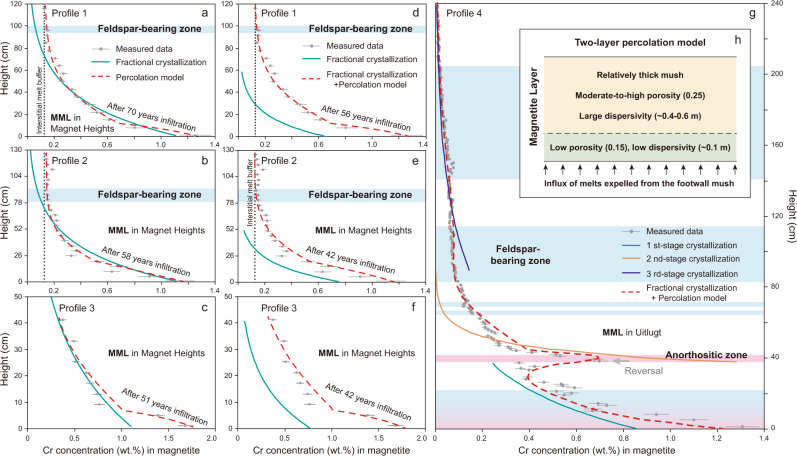
Vertical profiles of Cr content in magnetitites. Previously published measured vertical profiles (1–4) of Cr contents in magnetite from the MML compared with results of our models conducted using parameters summarized in Table 1. Solid lines in **a**–**c** show trends resulting from various fractional crystallization models chosen to match the main parts of the profiles as well as possible; these fail to match the upper and basal parts of the profiles. Red dashed lines in **a**–**c** also show the effects of reactive melt infiltration from below the MML after hypothetical complete homogenization of the magnetite by re-equilibration with the overlying magma. It is possible to mimic the effects of fractional crystallization purely by reactive melt infiltration through a homogeneous mush pile. Solid lines in **d**–**f** show fractional crystallization trends chosen to permit subsequent reactive melt infiltration to modify them and bring them into line with the observed profiles (red dashed lines). In **g** reactive infiltration is imposed on a primary stack of magnetite mush recording three distinct episodes of fractional crystallization, with the first at the bottom. **h** Diagram for the two-layer percolation model described in the text as reactive melt infiltration. All data were collected from multiple sources cited in the text, e.g., profiles in **a**, **b**, **d**, **e** ^22^, **c**, **f** ^18^ and **g** ^12^.

One deposit that has long been used as a laboratory to address the controls on layer formation in layered intrusions is the Main Magnetite Layer (MML) in the Upper Zone of the Rustenburg Layered Suite of the Bushveld Complex of South Africa^9–19^. The Upper Zone of the Bushveld Complex crystallized from an iron-rich basaltic andesite magma at the top of the Rustenburg Layered Suite in what appears to have been a melt-dominated sill hundreds of km wide and at least several hundred m deep^17^. The evolution of this magma and its cumulates can be modeled broadly as the product of a protracted process of fractional crystallization at the scale of the entire Upper Zone^3,17,20^, however, the Upper Zone contains at least 26 magnetitite layers that are ~0.1–10 m thick and concordant with the igneous layering. The formation of these magnetite layers cannot be accommodated by a simple process of fractional crystallization from a single homogeneous body of liquid the size of the entire Upper Zone. Instead, it seems probable that a repeated cyclic process of spontaneous double-diffusive convection occurred wherein the rejected liquid complementary to the gabbroic cumulates was denser than the main body of magma, causing the system to become stratified with a dense Fe-rich residual melt separating the growing cumulate pile from the rest of the magma^17,21^. It is thought that when magnetite became saturated and a layer of magnetitite was crystallized from this dense Fe-rich layer the liquid density rapidly diminished, causing convective overturn and re-homogenization of the magnetite-depleted basal layer with the rest of the magma and terminating magnetite crystallization until the closing stage of the next such cycle. Magnetitite layers generally have sharp and smooth or undulating boundaries with underlying anorthosites, but grade upwards into magnetite-rich gabbro due to smoothly increasing plagioclase modal abundance^13,17^. Along with abundant anorthosite xenoliths, narrow plagioclase-rich layers are widely developed within magnetitite layers (Fig. 1). The other portions are composed almost entirely of magnetite with minor interstitial plagioclase and pyroxene, effectively constituting monomineralic adcumulates. The economically important Main Magnetite Layer (MML), near the bottom of the Upper Zone, has ~1–2.5 m thickness and crops out intermittently over hundreds of km of strike extent^10–14,22^. It has been the focus of efforts to explain magnetitite formation.

The MML has a number of similarities with chromitites (they are both composed of spinels, are essentially monomineralic, associated with anorthosites, etc.) for which models range from orthomagmatic^4,23^ to suggestions that volatiles were important in their formation^24^. These hydromagmatic models have not yet been applied to the MML and here we look only at an orthomagmatic model. Although the origin of magnetitite layers has spurred an ongoing debate between various orthomagmatic hypotheses^18,22^, there has been general agreement that the magnetitite we observe now is an adcumulate evolved from a magnetite-dominated mush with a high initial amount of interstitial melt^13,25^. However, Kruger and Latypov^18^ recently proposed that this mushy zone had negligible thickness, being deposited directly onto a solid substrate in situ to form magnetite adcumulates with little or no initial interstitial melt. The highly compatible behavior of Cr in magnetite^26^ makes it sensitive to magmatic processes (e.g., crystallization and reactive melt infiltration), and the quasi-exponential vertically decreasing Cr gradients in monomineralic magnetitite layers have been widely attributed to fractional crystallization at or near the magma chamber floor^10–12,14,15,18^. Failure of a simple one-stage Rayleigh fractionation model to match observed compositional profiles (e.g., Fig. 1a–c) led to propositions that the supply of Cr was modulated by double-diffusive convection in overlying melt domains^10–12,14,15^. By repeatedly changing the Cr concentration in the melt, model trends could be produced that matched the data. Models of this sort can be shown to be consistent with observations but are not persuasive means to compare hypotheses when the boundary conditions are endlessly changeable, thus ensuring that a fit to the data can always be achieved.

Here we compare observations of the MML with some forward thermodynamic and fluid dynamic models, appealing to various combinations of fractional crystallization, compositional convection during adcumulus crystal growth, and reactive transport of upward-migrating Cr-rich melt from beneath the MML. By comparison of our models with previously published work, we show that multiple model approaches can achieve similar results, with the result that no model can be used definitively to exclude any other; however, some models are intrinsically more appealing than others because they require fewer arbitrary adjustments to achieve a successful fit to the data. In the immediate context of mechanisms of layer formation in layered intrusions, we show that purportedly incontrovertible evidence for in situ crystallization of magnetite can better be used to support a role for melt infiltration from below a magnetite crystal mush, but the choice of model remains a matter of preference.

## Results

### Modeling overview

We use several types of models to address the origins of compositional variation in the MML. First, we attempt to match observed profiles with simple models of fractional crystallization, finding that this is impossible without requiring some adjustments. Next, we address the effects of melt migration through a porous magnetite cumulate pile driven by density contrasts between residual silicate melt within the pile compared to the melt above it (i.e., *compositional convection*). To set up the models involving melt migration we must first explore the rate of diffusive re-equilibration of magnetite crystals to hypothetical changes in the composition of the interstitial liquid, and the time scale of compaction of a magnetite crystal mush in response to the density contrast between magnetite and interstitial melt. Combining these ideas, we examine the consequences of compaction-driven ascent of interstitial melts from the anorthositic footwall (i.e., *reactive melt infiltration*) in two distinct scenarios, one where the original record of fractional crystallization in the lower part of the profile has been erased by compositional convection (Fig. 1a–c), and another where it has not (Fig. 1d–h). Finally, we address lateral variations in magnetite composition with a 2-dimensional model of reactive melt infiltration into a pile of magnetite of uniform composition. The details of the model approaches are given in the “Methods” section, and their application is described in the following sections. No one model is meant to encompass the entire history of the compositional evolution of the MML; rather, they each explore the consequences of one or more processes in isolation or in combination in the generation of problematic features of the observed compositional profiles.

### Fractional crystallization models

Vertical profiles of Cr distribution in the MML, shown from Magnet Heights and Uitlugt in Fig. 1, commonly show a curvilinear upward depletion from the bottom, which broadly matches expectations based on the compatibility of Cr during fractional crystallization of magnetite^10–12^, but in each case, the predictions do not match the data exactly. The Cr content decreases markedly more steeply (i.e., more rapidly with height) at the base of every section, it locally shows extreme reversals (Fig. 1g), and it becomes constant at higher levels, none of which it could do during fractional crystallization unless the melt composition or basic physical parameters changed repeatedly during the process^14,15^. A further complicating matter is the common occurrence of exceptionally Cr-rich nodes decorating the basal contact of the MML, showing strong lateral as well as vertical compositional gradients^16,18,19^. These features have been taken as evidence that magnetite initially nucleated and crystallized around discrete nodes which coalesced as they grew outwards and upwards^16,18^.

We begin by addressing the profiles at Magnet Heights, shown in Fig. 1a–f. The more complex Uitlugt profile (Fig. 1g) will be addressed afterward. Here we assume that the MML is crystallized from a thick magma sheet, and that magnetite is the sole crystalline phase during the formation of the crystal mush of the MML (see “Methods” section). In Fig. 1a–c, we show models of fractional crystallization of magnetite from a homogeneous body of melt containing 57 ppm Cr^3,27^, with *f*_O2_ between zero and one log unit below the quartz-fayalite-magnetite oxygen buffer (i.e., −1 < ΔQFM < 0)^20,28,29^ using a magnetite-melt partition coefficient ${k}_{{{{{{\mathrm{D}}}}}}}^{{{{{{{\mathrm{mt}}}}}}}}$ of 200 at 1150 °C^14,26^. If the initial thickness of the overlying magma is set to 120 m as shown (Table 1), on the order of the vertical distance to the next magnetite layer up section^17,20,30^, then the exponential decrease in Cr between 10 and 40 cm is reproduced by the model but in the upper portions of all of the profiles the model predicts much greater depletion of Cr than is observed (Fig. 1a, b). Furthermore, there is a short interval at the base of the MML in which Cr decreases much faster than predicted by the model (Fig. 1a–c). A simple model of fractional crystallization alone therefore only matches a short interval of the observed profile and is inadequate. Like our predecessors^14,15^, we conclude that more complex processes have been at play.

**Table 1:** Parameters for the fractional crystallization and reactive melt infiltration models.

Panel	Profile	Initial Cr in magma (ppm)	Thickness of magma sheet (m)	Cr in upwelling melt (ppm)	Initial Cr in magnetite (ppm)	Duration (years)
a	1	57	120	73.5	1240	70
b	2	57	120	73.5	1240	58
c	3	57	120	108	1240	51
d	1	40	60	73.5	–	56
e	2	40	60	73.5	–	42
f	3	40	60	108	–	42
g	First	45	100	80	–	11
g	Second	84	25	80	–	11
g	Third	8	145	80	–	11

We propose two mechanisms to account for the departures of the Magnet Hill Cr profiles from patterns consistent solely with fractional crystallization, both contingent on the assumption that the magnetitite cumulate was initially solidified as a mush that retained some porosity occupied by interstitial melt^13,25^. This could be consistent either with in situ crystallization or settling of magnetite crystals formed by homogeneous nucleation. First, convection of intercumulus melt at the top of the pile (i.e., *compositional convection*)^31,32^ might have driven thorough re-equilibration of magnetite grains with the overlying melt body to homogenize the upper portions of the pile and generate the flat profiles above ~60–80 cm in all profiles (flat in the sense of changing slowly or not at all with height). Second, the expulsion of Cr-rich intercumulus melt from underlying anorthositic cumulates (i.e., *reactive melt infiltration*) might have allowed upgrading of Cr contents in the basal parts of the layer. Both processes depend on the rapid re-equilibration of magnetite crystals with percolating melt, which we address below after considering the effects of compaction of the mush.

### Compaction of magnetite mush

**Fig. 2:**
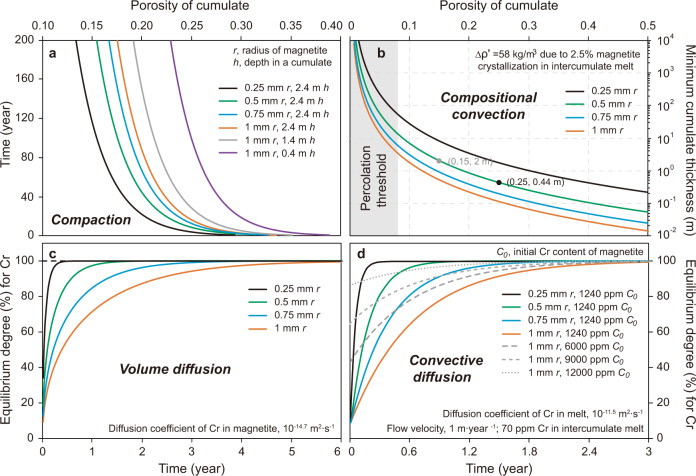
Model calculations of compaction and reactive transport in magnetite mush. Modeling results for **a** variation of magnetite mush porosity with time due to compaction. **b** Minimum thickness allowing compositional convection for mushes with indicated porosities. More thoroughly compacted lower portions of the mush may not spontaneously initiate compositional convection whereas more open upper portions could (Δ*ρ′* is density difference between interstitial melt and overlying melt in the main magma body). **c** Approach of the bulk composition of magnetite grains (integrated over the entire diffusion profile) during volume diffusion of Cr due to re-equilibration of magnetite with migrating intercumulus melt. **d** Approach of bulk composition to equilibrium via convective diffusion for various sizes and initial Cr contents *C*_0_ of magnetite. Colored lines are for crystal sizes as in **a**, **c**, and dashed lines represent magnetite grains with different initial Cr contents. Time scales of diffusive re-equilibration are much shorter than the time scale of compaction, supporting the assumption of instantaneous magnetite-melt equilibrium in the percolation model.

Isolated plagioclase crystals surrounded by magnetite record weaker deformation than clustered grains, suggesting that magnetite mush contained enough interstitial melt during early compaction to let the isolated crystals move or rotate freely^25^. Poikilitic pyroxene in magnetitite commonly encloses many separate and loosely packed subhedral–euhedral magnetite grains^13^; therefore, whether magnetite crystallized in situ or settled into place, there was initially substantial intercumulus melt. The sizes of the primary grains (~0.5–2 mm) are similar to that of disseminated magnetite in associated cumulate rocks, but far finer-grained than the crystals in silicate-free regions (~5–20 mm)^13^, indicating extensive overgrowth, compaction, and annealing to form the adcumulates. If it formed either by a process of fractional crystallization as a loose pile of settled crystals^3,4^ or as a loose arrangement of chains of magnetite crystals nucleated against others in situ^16,18,19^ and accumulated faster than the rate of compaction, the initial magnetite cumulate may have had a high porosity (~0.52)^33^. Compaction is driven by the large density contrast between solid (~5000 kg m^−3^) and liquid (~2700 kg m^−3^) (see “Methods” section). Generally, this process initiates with a mechanical reorganization to achieve optimum packing and continues with pressure solution/reprecipitation and possible viscous deformation of cumulate minerals^31,33–35^. The thickness of the MML (<~2.4 m) may not have provided sufficient gravitational load and effective stress to drive plastic deformation of magnetite^25^, and hence we argue that the compaction process was likely to have been achieved mainly through pressure solution and reprecipitation. On the basis of centrifuge experiments, Manoochehri and Schmidt^33^ quantified the compaction rate for a chromite cumulate in basaltic magma via dissolution-precipitation compaction. Given the similarity between chromite and magnetite, here we assume that the rate of chemical compaction for a magnetite mush layer is similar to that for chromite cumulate. The required time to reduce porosity from the initial value (~0.52, the average porosity after crystal settling)^33^ to about 0.25 in a magnetite cumulate is in the range from 10 to 40 years, or even less if crystals are smaller (Fig. 2a; see “Methods” for details of this calculation). We, therefore, conclude that the porosity of a magnetite pile one to two meters thick will mature over a decadal time scale to have low porosity similar to 0.25 at its base but will retain its primary porosity near 0.5 in its upper parts.

### Compositional convection and re-equilibration

While compaction is under way, continued crystallization of magnetite from intercumulus melt locally decreases the density of interstitial melt to a value lower than that of overlying magma, potentially causing it to rise as buoyant plumes into the overlying melt that must be convectively replaced by denser melt from the main magma body above the cumulate in the process of compositional convection^31,32^.

During this process, which occurs entirely within the interstitial liquid and does not disturb the integrity of the enclosing framework of solids, the interstitial melt is continuously expelled and replaced by the overlying melt from the main magma body, maintaining a near-constant composition for the pore melt buffered by continuous reaction with the overlying melt^31,36^. This process is implicit in Langmuir’s in situ fractional crystallization model^37^, but here we consider the limits on its occurrence more directly. The occurrence of significant compositional convection of interstitial melt within a mush requires that the dimensionless local solutal Rayleigh number (*R*_a_) exceeds a critical value of about 25^32,38^ and that the characteristic convective velocity is higher than the rate of crystal accumulation^31^. The latter requirement is easily achieved in deep and large intrusions (e.g., the Upper Zone of the Bushveld Complex) due to the small accumulation rate (~0.1–1 m year^−1^)^31^. Therefore, the minimum thickness of the magnetite cumulate pile to allow compositional convection to be operative can be estimated when the *R*_a_ value of porous medium equals the critical value (see “Methods” section). Figure 2b shows the minimum mush layer thickness to achieve the critical Rayleigh number permitting the onset of compositional convection for a range of grain sizes if the decrease of melt density is driven by the crystallization of a further 2.5 wt.% magnetite from the intercumulus melt. For example, convection of the interstitial melt begins when the thickness of an initial 1-mm-grained crystal mush exceeds ~0.44 m at 25% porosity (black dot in Fig. 2b), while a lower porosity (15%) will require a much thicker magnetite cumulate (~2 m, gray dot in Fig. 2b) for compositional convection. Compositional convection is therefore expected to operate in the loosely packed upper portion of the mush layer but will cease or may never commence in the lower portions once compaction has reduced porosity.

During compositional convection and percolation, the interstitial melt will continuously modify the compositions of packed magnetite grains. Previous investigations have identified occurrences of compositionally heterogenous magnetite grains, but the observed systematic zonations of Al and Mg are exclusively related to the exsolution of spinel phases in the sub-solidus stage^39^. Some fluctuations of Cr occur in the rims of magnetite grains in contact with ilmenite or plagioclase and can also be attributed to local sub-solidus re-equilibrium^39^. Observed lack of other types of zonation, therefore, indicates that if any compositional modification of primary magnetite grains was driven by the convection and percolation processes, they were likely to have been completed in the early stages of post-cumulate processes. Alternatively, it is possible that magnetite grains were homogeneous throughout their early history, only acquiring the limited observed zonation during cooling.

Next, we want to know whether diffusive re-equilibration of magnetite would occur rapidly enough that we can consider it to have been instantaneous in the context of melt percolation models. If re-equilibration between convecting interstitial melt and magnetite grains did occur, it would have involved two kinetic processes: the volume diffusion in single magnetite grains and the diffusion of elements through a viscous and diffusive boundary melt layer between the surrounding melt and magnetite (i.e., convective diffusion)^40^. The volume diffusion of Cr in a magnetite grain is simplified as the penetration diffusion of Cr into a spherical magnetite from an infinite source (i.e., interstitial melt)^41,42^ (see “Methods” section) and is controlled by *D*_Cr-mt_, the diffusion coefficient of Cr in magnetite. Note that here we use the parameter *D* to denote a diffusion coefficient, as opposed to the partition coefficient which here is written as ${k}_{{{{{{\mathrm{D}}}}}}}^{{{{{{{\mathrm{mt}}}}}}}}$. Due to the relative motion between flowing interstitial melt and magnetite, the supply of nutrient for magnetite growth is maintained fast enough to support our assumption of a constant boundary composition on the surfaces of magnetite crystals for the volume diffusion^43^. Our models for magnetite growth controlled by diffusion through a narrow boundary layer against a convecting melt^43^ and volume diffusion within solid magnetite^41,42^ confirm that Cr-poor grains (size < 1 cm) rapidly approach equilibrium with passing melt, and the required time is reduced with increasing initial Cr contents of the grains (Fig. 2c, d). Most magnetite grains would attain local equilibrium with the percolating melt in the post-cumulus stage, except for the retention of Cr gradients in rare coarse crystals (>1 cm)^39,44^. This would be further facilitated by recrystallization during extensive annealing and compaction^13,42^. We, therefore, assume that Cr content of magnetite in the dominant fine-grained population will be equilibrated with percolating melts.

Based on the Cr content of clinopyroxene in the closest hanging wall of the MML^29^, the overlying magma is proposed to have contained ~6.2 ppm Cr after the formation of the magnetite mush. Continuous loss of buoyant interstitial melt and its replacement by Cr-depleted melt containing ~6.2 ppm Cr would maintain the upper magnetite grains at a constant Cr content (~1240 ppm), which fits the profiles in Fig. 1a, b. Plagioclase can appear in the sequence without any correlation to the Cr trend (Fig. 1), because in this situation the Cr content of magnetite has become decoupled from its initial value, whereas plagioclase would resist diffusive re-equilibration because solid state diffusion is so slow in feldspar^45^. We conclude that flattening of the upper portions of the profiles could have resulted from compositional convection within the magnetite mush rather than requiring changes in the composition or depth of the magma column from which it initially crystallized (cf. McCarthy and Cawthorn^12^).

### Reactive melt infiltration

The footwall of the MML is proposed to have been permeated by interstitial melt that carried a slightly more primitive signature with it from lower parts of the UUMZ crystal pile, causing reverse zoning of cumulus plagioclase^21^ and crystallization of more anorthitic interstitial grains^25^. Whereas crystallization of magnetite from interstitial melt in the magnetitite could drive compositional convection, it is not expected during deposition of the anorthositic footwall when interstitial melt has density very close to that of the cumulus phase. However, sudden loading of the anorthosite pile by the deposition of a dense magnetite layer would tend to drive compaction and melt expulsion into the overlying magnetitite^46^. The slightly more primitive interstitial footwall melt would retain high Cr content because it had not yet equilibrated with magnetite in the footwall of the MML, further either enhanced by crystallization of Cr-free plagioclase in the footwall anorthositic mush or minimally diminished by the onset of crystallization of trace amounts of intercumulus magnetite. This Cr-rich melt would be discharged upward by the compaction of the footwall mush^30^. Hence, we presume an upward percolation of Cr-rich melt into the base of the recently formed magnetite mush, causing continuous modification of the Cr concentrations of magnetite grains. This process occurs simultaneously throughout the column of magnetite mush.

We solved this reactive transport problem using the Brinkman equations coupled with consideration of the dispersion around magnetite crystals via COMSOL Multiphysics for a two-dimensional system with a horizontal magnetite layer sitting above an anorthositic layer (see “Methods” section). The variation of porosity in the compacting magnetite cumulate as shown in Fig. 2a is simplified as two-layer model: a thin bottom zone (10 cm) with low porosity is overlain by thick mush with a higher porosity (Fig. 1h). In our model, a slightly Cr-enriched interstitial melt (~74–80 ppm Cr for the profiles 1–2 and 4 in Fig. 1a, b, d, e, g and Table 1) is expelled from underlying anorthositic mush at a compaction-driven velocity of ~1 m·year^−1^ ^31,34,35^. In Fig. 1a–c, we show what would happen if the percolation occurred into a magnetite pile that had been completely homogenized by compositional convection. In Fig. 1d–g, we show what would happen if the upper portions of the profiles had been flattened by compositional convection but their lower portions retained the signal of one or more episodes of fractional crystallization. The basal magnetite from the section in Fig. 1c contains a larger Cr content (~1.7 wt.%) and, accordingly, the magnetite-saturated melt expelled from footwall mush is assumed to have had a higher Cr content (~108 ppm, Table 1), which is equal to the Cr amount of the intruding parental magma above the Pyroxenite Marker^47^.

As a simple end-member scenario, the red lines in Fig. 1a–c show the results of the percolation model imposed on a pile of magnetite crystals along a one-dimensional profile through the top of one of the nodes in the two-dimensional model described below (Fig. 2). To identify better the influence of the percolation process, the magnetite grains in the whole cumulate pile are assumed initially to be equilibrium with a Cr-depleted, evolved melt (~6.2 ppm Cr), i.e., they initially contain a constant Cr content (~1240 ppm, Table 1) in the whole profile due to the effects of the compositional convection described above. Our percolation model trends account well for the steepening of Cr compositional gradients at the bottoms of all four one-dimensional sequences documented in Fig. 1, and the flat upper portions remain as imposed by reaction with the overlying melt due to compositional convection within the mush (Fig. 1a–c). Evidently, reactive melt infiltration alone is sufficient to account for the compositional variations generally attributed to fractional crystallization if we consider only the simple profiles observed at Magnet Heights. In Fig. 1d–g, we have first calculated Cr depletion trends due to fractional crystallization, then presumed that the uppermost portions of these profiles might have been flattened by compositional convection, and then applied the reactive melt infiltration model bringing up Cr-rich melt from the footwall. In these cases, because the melt coming in from the base is Cr-rich, the initial fractionated trends needed to be made rather lower in Cr to avoid overshooting the observed profiles. This was accomplished in Fig. 1d–f by assuming that magnetite was crystallized from a Cr-depleted melt (~40 ppm Cr) in a magma sheet with only 60 m thickness. The parameters for these and for the three stages of fractional crystallization in the Uitlugt profile in Fig. 1g are given in Table 1.

In summary, we have shown that reactive melt infiltration from below the MML can plausibly generate the observed shapes of the Cr-concentration profiles. This can be done either on a magnetite mush pile recording strong initial variations in Cr due to preservation of a record of single or multiple episodes of fractional crystallization, or in a mush pile that has been completely homogenized and re-equilibrated with the overlying melt by compositional convection prior to infiltration from below. The objective here is not to deduce exactly what happened using a single forward model, but rather to show that the general features of the profiles can be accounted for by some combination of fractional crystallization, compositional convection, and reactive melt infiltration by choosing suitable boundary conditions without the need to reset model parameters at every step in the calculations.

### Lateral variations in Cr concentration

**Fig. 3:**
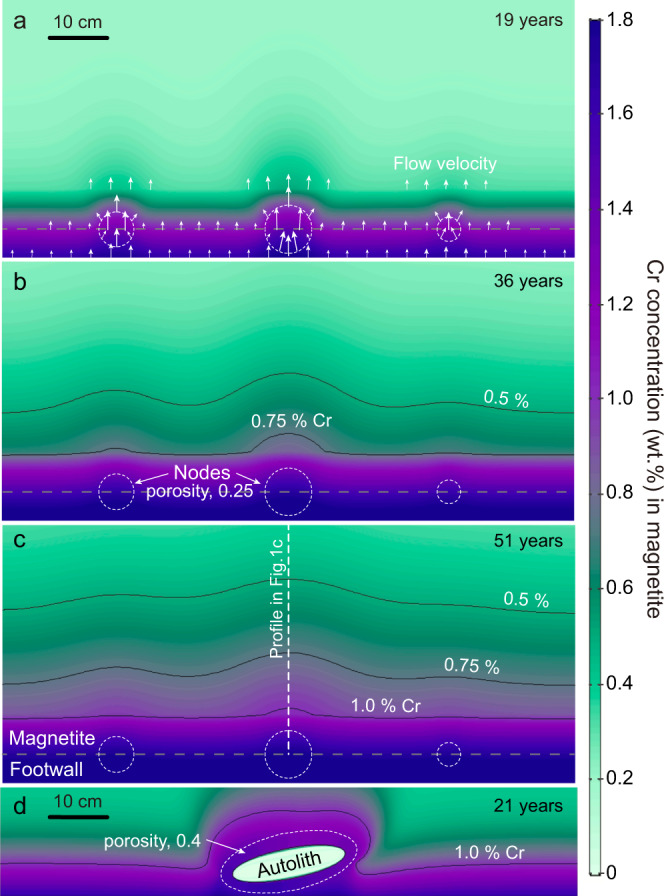
Two-dimensional models of reactive transport of Cr in magnetite crystal mush. **a**–**c** Snapshots of modeled Cr compositional mapping for the percolation of basal magnetite mush with three high-porosity spheric nodes (0.25, white dashed lines). White arrows in **a** plot for melt flow vectors. **d** Cr contours drape over the anorthosite autolith due to its high-porosity contact zone (0.4, white dashed line) with magnetite mush. Black lines represent Cr isopleths.

Others have inferred that concentration contours concentric around node-like structures at the base of the MML were caused by in situ growth that was initiated at discrete points on the floor^16,18,19^. Instead, we used our two-dimensional model of percolative reactive transport to address the alternative hypothesis that local variations in porosity in the underlying anorthosite mush may allow some sites to become preferred pathways for underlying melt during upward percolation. If Cr-rich melts flow through these nodes with a higher velocity and greater net mass flux, the grains here are able to extract more Cr than surrounding crystals, inducing lateral variations in Cr content along the base (Fig. 3a). Along with melt dispersion and upward flow in mush, this effect extends outwards to form undulations in Cr-concentration contours which gradually weaken over ~20–30 cm height (Fig. 3b, c), coinciding with the geochemical mapping on field outcrops of the MML^18^. We included an anorthosite autolith in the basal mush and considered that a solid block may impede early compaction of magnetite, leaving a high-porosity domain around the block which would enhance percolation. The resulting perturbation in the flow field led to concentric Cr enrichment around this autolith (Fig. 3d), producing spatial variations very similar to those shown by Kruger and Latypov^18,19^. This also partially accounts for the confusing phenomenon that Cr-rich growth nodes are found in the nooks beneath the autoliths but not directly on the upper surfaces of autoliths^18,19^.

### Multiple episodes of fractional crystallization combined with reactive transport

More complex profiles than those at Magnet Heights (Fig. 1a–f) require more complex adjustments to boundary conditions. We have combined the fractional crystallization model with the reactive transport model to address the complexity of the Uitlugt profile, which was sampled at 1 cm spacings. The large excursion in Cr content at 40 cm shown in Fig. 1g, which contains about 65% plagioclase, was reported not to be present in flanking boreholes by McCarthy and Cawthorn^12^, who attributed it to unspecified local convective fluctuations in magma composition during magnetite crystallization. To account for the evident presence of three segments of the profile, each decaying from progressively lower starting points, we must accept the possibility that convective overturn after deposition of the first ~40 cm of magnetite led to the start of a new episode of fractional crystallization from fresh Cr-rich melt, and a third such event occurred after deposition of about 90 cm of magnetite. However, we do not resort to a change in magma composition every time the profile departs from a perfect exponential decay. In Fig. 1g, we show the three initial profiles of Cr due purely to fractional crystallization, as well as the trend resulting from 11 years of the continued evolution of Cr due to percolative reactive transport of Cr-rich intercumulus melt from below (Table 1). The combined fractional crystallization and reactive transport model duplicates all the observed peaks and inflections in slope of Cr variation, using only three discrete crystallization sequences, for each of which the only adjustable parameter was the initial Cr content of the new magma.

## Discussion

Previous workers have inferred that the deposition of magnetite occurred in situ along the base of the magma chamber^10–12,16,18^. In these models there was no significant role for trapped liquid between magnetite crystals as they formed; lateral variations in Cr content were accounted for as resulting from initial magnetite nucleation at isolated growth centers that grew concentrically until they merged, and inflections in the slope of Cr concentration in vertical profiles were accounted for by numerous adjustments to the boundary conditions^16,18^. These adjustments varied from one model to the next, but all were designed to control the supply of Cr to the fractionating magnetite. In our models, magnetite is collected as discrete crystals to form an orthocumulate-textured mush during fractional crystallization; reversals of Cr gradients in more complex profiles are accounted for by as many as three episodes of convective overturn in the overlying magma (Fig. 1g). Percolation of melt through the mush could both have erased early compositional gradients in the upper portions of thick mushes and might also have allowed upward-migrating intercumulus liquid from below to steepen the Cr-concentration gradients near the base. Lateral variations in Cr concentration are accounted for by postulating that the porosity of the underlying anorthosite mush was not uniform; randomly distributed local enhancements in anorthosite permeability drove increased percolation at discrete locations in the base of the mush, generating concentrically-zoned domical patches of Cr enrichment (Fig. 3).

The difference between models of in situ magnetite crystallization and collection of loose crystals of magnetite may seem like a moot point^18^, but it drives at the difference between two fundamentally different mechanisms of layer formation^5^. If crystals in layered intrusions form within the melt and are transported to their eventual resting places, the implications for petrogenesis are dramatically different than they are if crystals form in situ on a hard substrate. A very wide range of possibilities is consistent with the deposition of a mush, but only one is permitted if crystallization proceeded in situ. It should be evident at this point that the match between observations and models afforded both by our models and by the previously published models places one in the uncomfortable position of having to choose between two very different sets of mechanisms to explain exactly the same observations with equal fidelity. In summary, all existing models, including ours, rely fundamentally on the idea that fractional crystallization of magnetite has generated the rapid and approximately exponential decay of Cr concentration in vertical profiles. The models differ in their explanations of inflections or reversals in the Cr profiles (Fig. 1) and of perturbations of Cr-concentration contours due to lateral variations (Fig. 3).

Geological observations can impose the primary criteria for testing these forward models. First, we consider whether it is more likely that magnetite arrived in its present location by in situ growth due to heterogeneous nucleation on a hard floor or by homogeneous nucleation in the melt followed by crystal settling to form a mush. The presence of plagioclase with magnetite in continuously variable proportions along with the abundance of autoliths is suggestive of co-accumulation of discrete grains due to crystal settling. The preservation of considerable relict intercumulus volume in pyroxene-poikilitic domains^13^ indicates that whether it occurred heterogeneously or homogeneously, magnetite crystallization initially led to formation of an open framework through which intercumulus melt would have been free to migrate before compaction and/or adcumulus growth brought porosity down below the percolation threshold (Fig. 2b)^31,48^. In situ crystallization experiments at low undercooling document the homogeneous nucleation of magnetite in basaltic melt even after initial heterogeneous nucleation on a solid substrate^49^. During diffusion-controlled growth, the preferred sites of incipient nucleation and rapid growth of magnetite would be expected to lie at the highest points on the surface rather than in deep reentrants under autoliths, regardless of their origins as relicts of eroded floor rocks^19^ or as xenoliths. Taken all together, these observations and interpretations alone do not allow a firm choice between homogeneous nucleation followed by crystal settling, or heterogeneous nucleation in situ at the magma chamber floor, but we lean towards a process dominated by homogeneous nucleation from the melt followed by settling of magnetite to form a highly permeable initial cumulate framework.

The choices of model parameters and boundary conditions may reflect the robustness and reliability of these forward models. The partition coefficient ${k}_{{{{{{\mathrm{D}}}}}}}^{{{{{{{\mathrm{mt}}}}}}}}$ of Cr between magnetite and melt is strongly controlled by oxygen fugacity (*f*_O2_) because Cr exists in both divalent and trivalent states over the range of *f*_O2_ commonly observed in natural silicate melts^26,50^. A choice of ${k}_{{{{{{\mathrm{D}}}}}}}^{{{{{{{\mathrm{mt}}}}}}}}$ of 525 by Kruger and Latypov^18^ was based on a highly-oxidized experiment (${k}_{{{{{{\mathrm{D}}}}}}}^{{{{{{{\mathrm{mt}}}}}}}}$ = 620 at ΔQFM > ~+4.9) conducted 45 years ago^51^, but recent experimental results have demonstrated a restricted range of ${k}_{{{{{{\mathrm{D}}}}}}}^{{{{{{{\mathrm{mt}}}}}}}}$ (<~300) for the common *f*_O2_ conditions (−2 < ΔQFM < +4.9) and the lowest ${k}_{{{{{{\mathrm{D}}}}}}}^{{{{{{{\mathrm{mt}}}}}}}}$ (~27–46) occurred in a oxidized system (ΔQFM, +3~+4.9)^26,52^. Here we have chosen a value of 200 which is consistent with *f*_O2_ within one log unit of QFM^20,26,28,29^. Previous models resorted to diffusive addition of Cr from convecting layers above^10–12,14,15,53^. Considering diffusive supply from a reservoir of fixed size to the crystallizing magnetite, Wright et al.^53^ and McCarthy et al.^14^ found that to fit the measured profiles for a fixed value of the Cr diffusion coefficient *D*_Cr-melt_ in the melt, the rate of crystal accumulation had to be arbitrarily constrained by the observed compositions. The rate of crystal accumulation implied by the model would require as much as 3 Myr to crystallize the 1 m thick MML^15^. Alternatively, Kruger and Smart^15^ showed that the profile could be fitted very well by assuming a continuous influx of Cr into the crystallizing layer of magma from an upper layer, above a stable diffusive boundary layer, in a stratified magma undergoing double-diffusive convection driven by crystallization at the base and heat loss out the top. However, their model could only be made to fit the observed compositional profiles by adopting extremely high and unrealistic values of *D*_Cr-melt_ of about 10^−9^ m^2^ s^−1^ whereas measured values in comparable andesitic melts are about 300 times smaller (3.2 × 10^−12^ m^2^ s^−1^ at 1150 °C)^54^. It is possible that by changing other model parameters their curves could be brought into line with measured data. The parameter least well constrained in their model is the thickness of the diffusive boundary layer separating the crystallizing lower layer from the layer above it. Since they never specified the value that they chose for the boundary layer thickness it is impossible to judge whether or not it could accommodate a change sufficient to match the data using correct values for *D*_Cr-melt_. In any case, both of these previous models work only if a key model parameter such as the thickness of a hypothetical diffusive boundary layer can be adjusted at will. Evidently, without independent evidence to guide the choices of such parameters, they can be dialed in to match any profile and the modeling process becomes an arbitrary exercise in curve-fitting.

Echoing the suggestion of Cawthorn^16^, Kruger and Latypov^18^ modeled solidification of the MML by in situ crystallization of magnetite directly attached to a solid substrate in a process of fractional crystallization from a well-mixed convecting reservoir of melt. As in other examples, their model would predict a continuous exponential decrease in Cr concentration and would fail to match the observed flattening of compositional profiles after the initial rapid decline (Fig. 1). To deal with this issue they added batches of fresh melt to their magma reservoir in amounts and with Cr contents arbitrarily chosen to force the model Cr trend to follow the observed trend. For example, Kruger and Latypov^18^ required successive injections of four discrete magma pulses with variable volumes to account for only the lowest 9 measurements in a 40-cm-thick profile (Fig. 1c, f). When a new pulse of magma is required in a model to account for every second data point, the model again becomes an arbitrary exercise in curve-fitting that loses predictive explanatory power. Furthermore, the two-dimensional model of magnetite crystallization had to use measured Cr-concentration contours to guide their modeled crystallization sequence^18^, rather than having the crystallization pattern governed by any physical model of diffusion-mediated crystal growth in a diffusive boundary layer.

There are weaknesses in our models as well. We have chosen boundary conditions and values for parameters like diffusivities and partition coefficients to match external constraints as well as we can; however, to match the complex Cr-concentration profile at Uitlugt we must resort to three separate magma recharge events timed to generate the observed inflections and we must perforce select a Cr content of the incoming melt to match the overall pattern. The principal differences between our models and previous ones are that we minimized the number of adjustable parameters (e.g., at most three magma influxes rather than one for every two data points, model melt compositions fixed by equilibrium with measured mineral compositions, use of experimentally measured compaction rates and diffusion coefficients) and we accounted for processes widely thought to have occurred in the Upper Zone such as post-cumulus melt migration and infiltration^25,30,55^ which were overlooked by previous approaches. The thickness of the melt layer from which the magnetite formed, which we and others have presumed to have been separated from the much deeper Upper Zone magma reservoir by a stable double-diffusive stratification, is another parameter that must be chosen arbitrarily. Values on the order of 60 to 120 m were chosen simply because they are probably in the correct order of magnitude, given that this is the typical separation between major magnetite layers^17,20,30^. Choice of a greater thickness would effectively diminish the rate of Cr depletion during fractional crystallization, a difference that could equally well be accommodated by diminishing the Cr partition coefficient.

Forward models of magmatic processes that cannot be directly observed can be valuable as descriptions of what could plausibly have happened, but they cannot be regarded uncritically as demonstrations of what really happened. When we are faced with multiple contradictory models that can adequately generate an acceptable quantitative match to observations, we must make our final choice based on the law of parsimony, also called Occam’s Razor—we favor the simplest hypothesis that requires the smallest number of artificially imposed external constraints while simultaneously acknowledging that we cannot be sure of the answer. A modeling scenario that can be set up to match the observations by setting nothing more than a plausible set of initial conditions and acceptable parameter choices is greatly preferable to another that requires continuous and arbitrary fine-tuning of model parameters to achieve a match to observations, even more so when the arbitrary models can only succeed through the adoption of physical parameters that are far outside of the ranges dictated by experimental observations.

In the balance, although both sets of models can be shown to work to explain the data, we prefer our model of melt migration and reaction with a magnetite crystal mush that was itself generated in some localities by multiple pulses of deposition from a magma sheet undergoing episodic replenishments due to convective overturn. The significance of the conclusion is that it speaks to a fundamental bifurcation in our understanding of the genesis of layered intrusions—do crystals deposited from magmas form in situ on solid substrates or are they formed in one place and deposited in another by transport and settling? These two concepts have dramatically important effects in controlling how we understand the formation of layered intrusions in general and, more specifically, the genesis of critically important mineral deposits of Fe, Ti, V, Ni, Cu, Co, Pt, Pd, etc. The importance of in situ crystal growth is not unequivocally established by observations of the MML; to the contrary, our work makes it seem much more likely that the MML was deposited as a mush. Both the in situ and mush origin hypotheses are falsifiable and hence fall within the realm of Popperian science. Firm evidence that could falsify the in situ hypothesis might be found in primocrysts preserved as floating grains of magnetite not physically in contact with other magnetite grains within pyroxene oikocrysts (Fig. 1c, e of Reynolds^13^), however, without three-dimensional imaging, it cannot be proven that these grains truly were isolated from others and therefore did not grow in situ attached to a solid substrate. What we have shown is that although it is still allowable there is little evidence in support of the in situ hypothesis and the evidence offered in its support may better support the mushy origins of the MML. Evidently, our current knowledge of the Main Magnetite Layer does not, as yet, carry the information we need to make a definitive choice, and it is important for us to remember that as we continue to search for more definitive answers to a perplexing problem at the heart of igneous petrology.

## Methods

### Analytical data

The analytical methods for the data presented were given by the original authors^12,18,22^. For profiles 1–2 and 4 in Fig. 1, the samples were crushed and screened to small mesh, and magnetically separated by hand magnet. Pressed pellets of pure finely pulverized magnetite were analyzed by X-ray fluorescence (XRF) spectrometry. The profile 3 in Fig. 1 was obtained by portable XRF analysis in the field. These XRF data were calibrated by the in-house XRF analysis on pure magnetite separates from the MML, which is the same as the Cr measurements for other profiles in Fig. 1. The variation of modal abundances of phases other than magnetite has no effect on the measured Cr concentrations.

### Equations for fractional crystallization model

In each increment of the fractional crystallization, the conservation of Cr mass in a closed system requires that:

1 $${C}_{i-1}^{{{{{{{\mathrm{melt}}}}}}}}\cdot {H}_{i-1}\cdot {\rho }_{i-1}^{{{{{{{\mathrm{melt}}}}}}}}={C}_{i}^{{{{{{{\mathrm{melt}}}}}}}}\cdot {k}_{{{{{{\mathrm{D}}}}}}}^{{{{{{{\mathrm{mt}}}}}}}}\cdot \left({H}_{i-1}-{H}_{i}\right)\cdot {\rho }_{{{{{{{\mathrm{mt}}}}}}}}+{C}_{i}^{{{{{{{\mathrm{mt}}}}}}}}\cdot {H}_{i}\cdot {\rho }_{i}^{{{{{{{\mathrm{melt}}}}}}}}$$

where ${C}_{i}^{{{{{{{\mathrm{melt}}}}}}}}$, ${H}_{i}$ and ${\rho }_{i}^{{{{{{{\mathrm{melt}}}}}}}}$ are the Cr content, thickness, and density of melt at *i-*th increment, respectively, and the ${k}_{{{{{{\mathrm{D}}}}}}}^{{{{{{{\mathrm{mt}}}}}}}}$ is the partition coefficient of Cr between magnetite and melt, and here is assumed as 200 based on recent experimental measurements^26^. The density of magnetite (${\rho }_{{{{{{{\mathrm{mt}}}}}}}}$) is estimated as ~5000 kg m^−3^ ^56^. The melt density keeps decreasing due to the crystallization of denser magnetite phase, and if we make the simplifying assumption that the partial molar volume of the Fe_3_O_4_ component in the melt is the same as that of magnetite, its value at the *i-*th increment can be calculated as:

2 $${\rho }_{i}^{{{{{{\mathrm{melt}}}}}}}=\frac{{\rho }_{0}\cdot {H}_{0}-{\rho }_{{{{{{{\mathrm{mt}}}}}}}}\cdot ({H}_{0}-{H}_{i})}{{H}_{i}}$$

where ${\rho }_{0}$ is the density of initial melt (~2700 kg m^−3^), and *H*_0_ is the initial thickness of melt. The thickness of the magnetite layer at the *i-*th increment is expressed as (*H*_0_ − *H*_*i*_), and the Cr concentration of crystallized magnetite within this increment (${C}_{i}^{{{{{{{\mathrm{mt}}}}}}}}$) is calculated as:

3 $${C}_{i}^{{{{{{{\mathrm{mt}}}}}}}}={C}_{i}^{{{{{{{\mathrm{melt}}}}}}}}\cdot {k}_{{{{{{\mathrm{D}}}}}}}^{{{{{{{\mathrm{mt}}}}}}}}$$

### Compaction time calculation

The compaction time (*t*) is the required time to reduce porosity from the initial porosity (~0.52) to a given lower value in the basal layer of a magnetite cumulate, and can be calculated as^33^:

4 $$t=\frac{5.471\times {10}^{18}}{{10}^{(14.35\cdot \varphi )}}\,\times \frac{d}{h\cdot \triangle \rho }$$

where is *φ* the porosity of cumulate pile, *d* is the diameter of magnetite grains (m), *h* is the depth in crystal mush (m), and $\triangle$*ρ* is the density difference between the magnetite and interstitial melt (kg m^−3^). On the basis of our fractional crystallization model^3^, the density of the evolved melt that corresponds to the position of the MML in the Upper Zone can be estimated as ~2700 kg m^−3^ via the parameterized equation from Lesher and Spera^57^.

### Compositional convection model

Based on the critical value of the Rayleigh number (*R*_a_, ~25)^32,38^, the minimum thickness (*h*_min_) of the magnetite cumulate pile allowing compositional convection to be operative can be estimated as^31^:

5 $${h}_{{\min }}=\frac{{R}_{{{{{{\mathrm{a}}}}}}}\cdot {\mu }_{{{{{{\mathrm{m}}}}}}}\cdot {D}_{{{{{{{\mathrm{Cr}}}}}}}{-}{{{{{{\mathrm{melt}}}}}}}}}{k\cdot g\cdot \triangle {\rho }^{{\prime} }}$$

where *μ*_m_ is the melt viscosity (~160 Pa s)^3,58^, *D*_Cr-melt_ is the effective chemical diffusivity in the melt, *k* is the permeability of crystal mush, *g* is the gravitational acceleration, and $\triangle$*ρ*′ is the density difference between the interstitial melt and overlying magma. The diffusion coefficient of Cr in melt is estimated as 3.2$\times$10^−12^ m^2^ s^−1^ ^54^, and adopted as the *D*_Cr-melt_ here. The decrease of melt density driven by the crystallization of 2.5 wt.% magnetite from the pore melt is calculated as 58 kg m^−3^, which can be considered as the $\triangle$*ρ*′. At a high porosity, the permeability of crystal mush is simplified as^35,59,60^:

6 $$k={d}^{2}\cdot {\varphi }^{3}/300$$

where *d* is the diameter of magnetite grains and *φ* is the porosity of crystal mush.

### Equation for the diffusion model

For the bulk diffusion of Cr in a spherical magnetite grain with uniform initial composition and a boundary set to a different fixed composition by reaction with an infinite surrounding medium, the equilibrium degree of Cr can be expressed as the ratio of *M*_*t*_ (total amount of diffusing Cr entering or leaving the magnetite at time *t*) to ${M}_{\infty }$ (corresponds the amount of diffusing Cr after infinite time to reach equilibrium)^41^:

7 $$\frac{{M}_{{{{{{\mathrm{t}}}}}}}}{{M}_{{{\infty }}}}=1-\frac{6}{{\pi }^{2}}\mathop{\sum }\limits_{n=1}^{{{\infty }}}\frac{1}{{n}^{2}}\cdot {e}^{-{D}_{{{{{{{\mathrm{Cr}}}}}}}-{{{{{{\mathrm{mt}}}}}}}}\cdot {n}^{2}\cdot {\pi }^{2}t/{r}^{2}}$$

where *r* is the radius of magnetite grain, *t* is the time and *D*_Cr-mt_ is the diffusion coefficient of Cr in magnetite (~2 × 10^−15^ m^2^ s^−1^)^26,40^. On the other hand, during the convective diffusion, a compositional gradient of Cr persists within a thin diffusive boundary layer around the magnetite grain, and the mass transfer (d*M*_Cr_) of an element Cr via this compositional boundary layer within a short time interval (d*t*) can be approximated as^43^:

8 $$\frac{{{{{{\mathrm{d}}}}}}{M}_{{{{{{{\mathrm{Cr}}}}}}}}}{{{{{{{\mathrm{d}}}}}}t}}=4\pi {r}^{2}{\rho }_{{{{{{\mathrm{m}}}}}}}\cdot {D}_{{{{{{{\mathrm{Cr}}}}}}}{-}{{{{{{\mathrm{melt}}}}}}}}\cdot \frac{{C}_{{{{{{\mathrm{m}}}}}}}-{C}_{0}}{{\delta }_{{{{{{{\mathrm{Cr}}}}}}}}}$$

where *r* is the magnetite radius, *ρ*_m_ is the density of melt (~2700 kg m^-3^), *D*_Cr-melt_ is the diffusion coefficient of Cr in melt (~3.2 × 10^−^^12^ m^2^ s^−^^1^)^54^, *C*_m_ is the Cr content of interstitial melt, *C*_0_ is the initial Cr concentration of magnetite, and *δ*_Cr_ is the boundary layer thickness for Cr. The Cr flux predicted by this equation is summed for successive time intervals to track the compositional variations of magnetite during this convective diffusion. The *δ*_Cr_ is mostly controlled by the strength of convection, and can be expressed as^61^:

9 $${\delta }_{{{{{{{\mathrm{Cr}}}}}}}}=2r/\left[1+{(1+\frac{2{rV}}{{D}_{{{{{{{\mathrm{Cr}}}}}}}-{{{{{{\mathrm{melt}}}}}}}}})}^{1/3}\right]$$

where *V* is the flow rate of interstitial melt (~1 m year^−^^1^)^31,34,35^.

### Reactive melt infiltration model

The Brinkman equations extend Darcy’s law to model the dissipation of kinetic energy by viscous shear, which is similar to the Navier–Stokes equations, and are also connected to a set of reaction–diffusion–convection equations. Here, the Brinkman equations for steady-state flow are:

10 $$\left\{-\nabla \cdot \frac{\mu }{\varphi }\left[\nabla {{{{{\bf{u}}}}}}+{(\nabla {{{{{\bf{u}}}}}})}^{T}\right]\right\}-\left(\frac{\mu }{k}\cdot {{{{{\bf{u}}}}}}+\nabla p-{{{{{\bf{F}}}}}}\right)=0\,\,{{{{{\rm{and}}}}}}\,\,\nabla \cdot {{{{{\bf{u}}}}}}=0$$

where *μ* is melt viscosity, **u** represents the velocity vector, *p* is pressure, *φ* is the porosity and *k* denotes the permeability of crystal mush. Gravity is also included in this model, and its influence is accounted for by the force term, **F**. The grain size of magnetite is assumed as 2 mm in the model^13^, and the relation between porosity and permeability follows the Kozeny–Carman model^62^. The transport of solute Cr in the migrating melt due to liquid dispersion, advection, and molecular diffusion is modeled via the advection–dispersion equation:

11 $$\varphi \frac{\partial \gamma }{\partial t}+\nabla \cdot \left(-\varphi \cdot {{{{{{\bf{D}}}}}}}_{{{{{{\mathrm{L}}}}}}}\cdot \nabla \gamma +{{{{{\bf{u}}}}}}\gamma \right)={S}_{{{{{{\mathrm{C}}}}}}}$$

where *φ* is the porosity, *γ* is the mass concentration of Cr in melt (kg m^−3^), **u** is the flow velocity (m s^−1^), **D**_L_ is the hydrodynamic dispersion tensor (m^2^ s^−^^1^), and *S*_C_ represents the quantity of Cr added per unit volume of porous medium per unit time at the bottom of magnetite cumulate pile (kg m^−3^ s^−^^1^). The hydrodynamic dispersion tensor (**D**_L_) describes the combined influences of the chemical diffusivity in the melt and the mechanical dispersivity via grain-scale flow in porous media. Hence, the diagonal components of the **D**_L_ are controlled by the diffusion coefficient of Cr in the andesitic melt (*D*_Cr-melt_, ~ 3.2 × 10^−^^12^ m^2^ s^−^^1^)^54^, and the dispersivities along the longitudinal and transverse flows. Previous work demonstrated a systematic increase of the longitudinal dispersivity with the corresponding observational scale of porous media^63^, and we assume ~0.1 and ~0.4–0.6 m for the two layers in modeled mush, respectively (Fig. 1h). The transverse dispersivity in experiments is typically an order of magnitude smaller than that of longitudinal dispersivity^64^, which is also adopted here.

## Data Availability

All data shown here were obtained from the cited references.
